# Neurogenic Inflammation of Guinea-Pig Bladder

**DOI:** 10.1155/S0962935194000268

**Published:** 1994

**Authors:** D. E. Bjorling, M. R. Saban, R. Saban

**Affiliations:** Smooth Muscle Laboratory Department of Surgical Sciences School of Veterinary Medicine University of Wisconsin Madison WI USA

## Abstract

Capsaicin, substance P, and ovalbumin, instilled into the bladders of naive and ovalbumin (OVA) sensitized guineapigs caused inflammation, as indicated by increased vascular permeability. Histological changes after exposure to these compounds progressed with time from intense vasodilatation to marginalization of granulocytes followed by interstitial migration of leukocytes. *In vitro* incubation of guinea-pig bladder tissue with substance P and ovalbumin stimulated release of prostaglandin D_2_ and leukotrienes. *In vitro* incubation of bladder tissue with capsaicin, OVA, prostaglandin D_2_, leukotriene C_4_, histamine, or calcium ionophore A-23587 all stimulated substance P release. These data suggest that bladder inflammation initiated by a variety of stimuli could lead to a cyclic pattern of release of inflammatory mediators and neuropeptides, which could result in amplification and persistence of cystitis after the inciting cause has subsided.


Mediators of Inflammation 3, 189–197 (1994)

### Neurogenic inflammation of guinea-pig bladder

D. E. Bjorling,[CA] M. R. Saban and R. Saban

Smooth Muscle Laboratory, Department of Surgical Sciences, School of Veterinary Medicine, University of Wisconsin, Madison, WI, USA

[CA] Corresponding Author

**Capsaicin, substance P, and ovalbumin, instilled into the bladders of naive and ovalbumin (OVA) sensitized guinea-pigs caused inflammation, as indicated by increased vascular permeability. Histological changes after exposure to these compounds progressed with time from intense vasodilatation to marginalization of granulocytes followed by interstitial migration of leukocytes. *In vitro* incubation of guinea-pig bladder tissue with substance P and ovalbumin stimulated release of prostaglandin $D_2$ and leukotrienes. *In vitro* incubation of bladder tissue with capsaicin, OVA, prostaglandin $D_2$, leukotriene $C_4$, histamine, or calcium ionophore A-23587 all stimulated substance P release. These data suggest that bladder inflammation initiated by a variety of stimuli could lead to a cyclic pattern of release of inflammatory mediators and neuropeptides, which could result in amplification and persistence of cystitis after the inciting cause has subsided.**

**Key words:** Antigen challenge, Bladder, Cystitis, Guinea-pig, Neuropeptides

### Introduction

Apparently spontaneous inflammation of the urinary bladder in the absence of infection has been reported in humans and cats.[1,2] In humans, the most widely recognized form of cystitis in the absence of infection is interstitial cystitis (IC), a chronic inflammatory disease of the lower urinary tract. Clinical symptoms commonly associated with IC include increased urinary frequency and urgency, nocturia, suprapubic pressure and pain.[1,3]

Although numerous theories regarding the aetiology of IC have been proposed, the pathogenesis remains unknown.[4] Traumatic injury, lymphatic obstruction and autoimmune disease are among the suggested causes.[5] Regardless of the cause(s) of IC and other forms of non-infectious cystitis, a number of reports suggest that the nervous system is intimately involved in the development and persistence of non-infectious cystitis.[5–7]

Neurogenic inflammation affecting various organs, including the skin, lungs, airways, gut, eye and joints, has been reported in several species, including humans.[8–10] A variety of stimuli, such as antigens, heat, cold, bacterial or viral infection, and direct stimulation of sensory nerves, can trigger the onset of neurogenic inflammation.[8–10] Neuropeptides such as substance P (SP), neurokinin A (NKA), calcitonin gene-related peptide (CGRP) and vasoactive intestinal peptide (VIP) are located in capsaicin sensitive primary afferent nerves in the bladders of humans and animals.[11,12] The release of SP, NKA and CGRP has been correlated with smooth muscle contraction, vasodilatation, increased vascular permeability and facilitated neurotransmitter release from intramural nerves.[13] SP, CGRP and VIP also mediate the sensory function of capsaicin sensitive nervous control of the micturition threshold and may facilitate spontaneous bladder contraction.[14] Experimentally, antidromic electrical stimulation of unmyelinated afferent nerve fibres causes extravasation of Evans blue dye in the urinary bladder of guinea-pigs.[15] Of the neuropeptides present in the afferent sensory nerve fibres, SP is thought to be the primary neurotransmitter responsible for initiation of neurogenic inflammation, and CGRP is believed to play an important role in facilitating and mediating the effects of SP.[16] Intravenous administration of SP also causes extravasation of Evans blue dye and accumulation of leukocytes in the bladder wall of experimental animals, and intravesical application of SP has a similar effect.[17,18]

Capsaicin, an extract of hot pepper, is a neurotoxin for unmyelinated sensory nerve fibres which selectively depletes SP and other neuropeptides from the nerves at low doses and causes degeneration of these nerves at high doses. Pretreatment of rats with capsaicin significantly reduced the severity of xylene induced cystitis, strongly suggesting the participation of SP in the pathogenesis of this model of cystitis.[19]

It has been reported that SP stimulates histamine release from various tissues and isolated mast cells, but this effect appears to be specific to the tissue source of the mast cells and the species from which these tissues and mast cells are obtained.[20] Preliminary studies in our laboratory have indicated that

© 1994 Rapid Communications of Oxford Ltd isolated guinea-pig bladder tissue releases histamine in response to exposure to capsaicin or SP. We have previously described the results of investigations using a guinea-pig model for IC in which cystitis develops in response to antigen sensitization and challenge.[21,22] Antigen challenge of sensitized bladder tissue in this model causes release of histamine, prostaglandins and leukotrienes, and this model of cystitis was used to test the hypothesis that antigen challenge of sensitized guinea-pig urinary bladder tissue would stimulate SP release from sensory afferent nerves and that exposure of guinea-pig bladder tissue to inflammatory mediators would also stimulate release of SP from sensory afferent nerves, suggesting a positive feedback mechanism for the amplification of cystitis.

### Materials and Methods

Female, albino guinea-pigs (450–600 g) were used in these experiments. Sensitized animals were actively sensitized to ovalbumin (OVA; chicken egg albumin, ovalbumin grade V; Sigma Chemical Company, St Louis, MO) by administering three intraperitoneal injections of OVA (10 mg/kg) at 48 h intervals. Control animals received injections of the same volume of saline at the same time intervals. Twenty-one days after the last injection, animals were sacrificed for *in vitro* studies with sodium pentobarbital (100 mg/kg, i.p.) or anaesthetized for *in vivo* studies as described subsequently. For *in vitro* studies, urinary bladders were removed and placed in physiologic salt solution (PSS) of the following composition (mM): NaCl, 119; $NaH_2PO_4$, 1; KCl, 4.7; $CaCl_2$, 2.5; $MgCl_2$, 0.5; $NaHCO_3$, 25; and glucose, 11. The PSS was maintained at 37 °C and aerated continuously with a mixture of 95% $O_2$ and 5% $CO_2$ (pH 7.4).

*Intravesical instillation of capsaicin, SP and OVA:* Guinea-pigs were anaesthetized with ketamine HCl (40 mg/kg, i.p.) and xylazine (2.5 mg/kg, i.m.). A polypropylene catheter with a closed end and side openings (3½ French Tom Cat Catheter, Sherwood Medical, St Louis, MO) was introduced transurethrally into the bladder. The catheter was advanced until the first drop of urine appeared. Urine was drained from the bladder by applying light pressure on the abdomen, and 3 ml of 0.9% saline, 3 μM capsaicin, 100 μM SP, or OVA (1 μg/ml) was infused. To ensure consistent contact of the bladder with saline or the test substances, the animals received a total of four instillations during a 2 h period. Some guinea-pigs were sacrificed by pentobarbital overdose 30 min after the first instillation, and the remainder were sacrificed 2, 4, 8 and 20 h after the first instillation. Bladders were removed and processed for histology.

Tissues were fixed in neutral buffered formalin, and 5 μm thick sections were stained with haematoxylin and eosin or Giemsa. Stained tissue sections were viewed and photographed using light microscopy. Microscopic images were scanned (Coolscan™, Nikon Electronic Images, Melville, NY) using Adobe Photoshop™ 2.5.1 software (Adobe Systems, Inc., Mountain View, CA) and printed with a postscript imager (Lynotronic™ Model 300, Lynotype, Hell, Germany) at 2 540 dots per inch.

*Measurement of plasma extravasation:* Plasma extravasation was quantified using a standard Evans blue dye technique.[15] Briefly, Evans blue dye (Sigma Chemical Company, St Louis, MO) was diluted in 0.9% saline (30 mg/ml), filtered with a 5.0 μm filter, and injected (30 mg/kg) into a jugular vein 15 min before intravesical instillation of saline, capsaicin, SP or OVA. Guinea-pigs were sacrificed 4 or 18 to 20 h after intravesical instillation of saline, capsaicin, SP or OVA for determination of plasma extravasation. Immediately prior to sacrifice, the thorax was opened, and a blunt 13-gauge needle was passed into the aorta via a left ventriculotomy. The right atrium was incised to allow outflow of perfusate, and the animal was perfused with 100 ml of 0.9% saline at 100 mmHg pressure to remove intravascular dye. The urinary bladder was removed, blotted three times on filter paper and weighed. Tissues were incubated in 2 ml of 100% formamide (Sigma Chemical Company, St Louis, MO) at 37 °C for 20 h to extract Evans blue dye, and the dye concentration was determined spectrophotometrically by light absorbance at 620 nm. Tissue concentrations of dye were expressed as ng dye per mg of wet tissue weight.

*Release of inflammatory mediators from bladder tissue:* Tissues were placed in individual test tubes containing 2 ml of PSS (37 °C) aerated with a mixture of 95% $O_2$ and 5% $CO_2$. The PSS bathing the bladder tissues in the test tubes was replaced with fresh solution at 15 min intervals for 60 min. After 60 min, tissues were incubated in fresh PSS for an additional 15 min, and this fluid was saved for subsequent determination of spontaneous release of leukotrienes, prostaglandin $D_2$ ($PGD_2$) or SP.

Leukotriene and $PGD_2$ release from bladder tissue incubated with SP was compared with release of these compounds stimulated by sensitized tissues exposed to OVA. Bladder tissue was incubated with OVA (1 μg/ml) or SP (100 μM) for 30 min, and the bath solution was collected for determination of concentrations of $PGD_2$ and leukotrienes.

To compare the capacity of various substances to stimulate SP release from the bladder, other tissues were challenged with capsaicin (3 μM), leukotriene $C_4$ ($LTC_4$, 100 μM), $PGD_2$ (100 μM), histamine (100 μM), OVA (1 μg/ml), or calcium ionophore A23587 (100 μM) for 30 min, after which the bath solution was collected for subsequent determination of SP release.

*$PGD_2$ radioimmunoassay:* $PGD_2$ content was determined by radioimmunoassay as described previously.[23] This assay is sensitive to about 0.03 pmol/0.1 ml.

*Leukotriene radioimmunoassay:* The amount of leukotriene released was measured by radioimmunoassay.[24] The limit of sensitivity of this assay is approximately 0.03 pmol as defined by that amount required to inhibit [$^3$H]-$LTC_4$ binding by 10%. The anti-peptidoleukotriene antibody is highly selective with little affinity (cross-reactivity < 1%) for a variety of heterologous eicosanoids. The antibody does not, however, distinguish markedly between $LTC_4$, $LTD_4$ and $LTE_4$. Therefore, the results are presented as leukotriene-like immunoreactivity (LLI).

*Substance P radioimmunoassay:* SP release was determined by radioimmunoassay (RIA). Five μg of synthetic [Tyr-O]-substance P was dissolved in 50 μl 0.1 M phosphate buffer, pH 7.5. One mCi (5 μl) of high specific activity $^{125}$I and 20 μl (20 μg) chloramine-T was added, and the mixture was agitated for 45 s. The reaction was stopped by addition of 200 μl saturated L-tyrosine. The labelled peptide was purified by gel filtration on a 0.9 × 30 cm column of G-35 Sephadex (fine) eluted with 0.02 M phosphate buffered saline, pH 7.5. [$^{125}$I]-O-Tyr-SP was nearly 100% bindable with excess antibody. Standards (0 to 2 000 pg/ml) or sample (200 μl) were added to 500 μl of anti-substance P (produced by G. P. Kozlowski; 1 : 8 000 initial dilution) in 0.1 M tris, pH 7.5 containing 0.5% normal rabbit serum. Tracer SP (100 μl containing 10 000 cpm) was added to each tube and mixed. Tubes containing only tracer, as well as non-specific binding tubes containing PBS–NRS buffer without antibody, were also prepared at this time. Samples were mixed and incubated for 24 h at 4 °C. Bound tracer was precipitated by addition of 100 μl of 1 : 4 sheep anti-rabbit IgG (produced in our laboratory) and 1 000 μl of 3.3% polyethylene glycol in PBS, and samples were centrifuged at 3 000 × *g* for 20 min. The supernatant was discarded, and bound radioactivity was counted in the pellet with a gamma counter equipped with RIA data reduction (Gammatrac Model 1290, Tm Analytic, Inc., Elk Grove Village, IL). Characteristics of this assay include a minimum detectable dose less than 10 pg/tube at $EC_{50}$, intra-assay variation less than 5%, and inter-assay variation of approximately 8%. Cross-reactivity studies (at $EC_{50}$) showed 100% cross-reactivity for SP, SP (4–11) and physalaemin, and less than 0.01% for metenkephalin and somatostatin. Cross-reactivity of other peptides is being evaluated.

Substance P degradation in samples was prevented by addition of a combination of peptidase inhibitors, including captopril ($5 \times 10^{-6}$ M), thiorphan ($1 \times 10^{-6}$ M), and phosphoramidon ($5 \times 10^{-6}$ M). SP was prepared in sodium metabisulphate (0.05% in saline) containing these inhibitors. In experiments investigating OVA-induced SP release, the PSS used contained these compounds.

*Statistical analysis:* Statistical analysis of the results was performed with Minitab™ 8.0 (Minitab Inc., Rosemont, PA) software using analysis of variance and Student's *t*-test for paired or unpaired data.[25] The *n* values refer to one strip of tissue per experimental animal. All values were expressed as mean ± standard error of the mean (S.E.M.), and a value of $p < 0.05$ was considered significant.

### Results

*Plasma extravasation:* Intravesical instillation of saline in control and sensitized animals, and OVA in non-sensitized animals, failed to stimulate detectable Evans blue dye extravasation. Four h after intravesical instillation of capsaicin (3 μM), SP (100 μM), or OVA (1 μg/ml, in sensitized animals), Evans blue dye extravasation had occurred (Fig. 1), and Evans blue dye extravasation was not increased when measured 18 to 20 h after intravesical instillation of these compounds. In preliminary studies, extravasation of Evans blue dye in response to 1 μg/ml OVA (the concentration of OVA used consistently in this model of cystitis[21,22]) instillation into sensitized bladders was determined, and extravasation of Evans blue dye in response to various concentrations of intravesical capsaicin and SP was investigated. The concentrations of capsaicin (3 μM) and SP (100 μM)

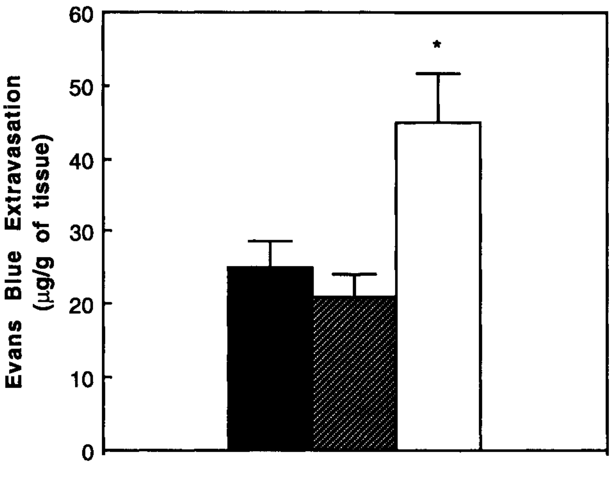

FIG. 1. Evans blue dye extravasation in guinea-pig bladders 4 h after *in vivo* intravesical infusion of ovalbumin (1 μg/ml, sensitized animals only), substance P (100 μM), or capsaicin (3 μM). Bladders in sensitized and naive guinea-pigs infused with saline contained 2–5 μg Evans blue dye/g tissue ($n = 12$; data not shown). Asterisk indicates significant difference ($p < 0.05$) between the effects of capsaicin ($n = 10$) and substance P ($n = 8$) or ovalbumin ($n = 8$). ■, ovalbumin, 1 μg/ml; ▨, substance P, 100 μM; □, capsaicin, 3 μM.

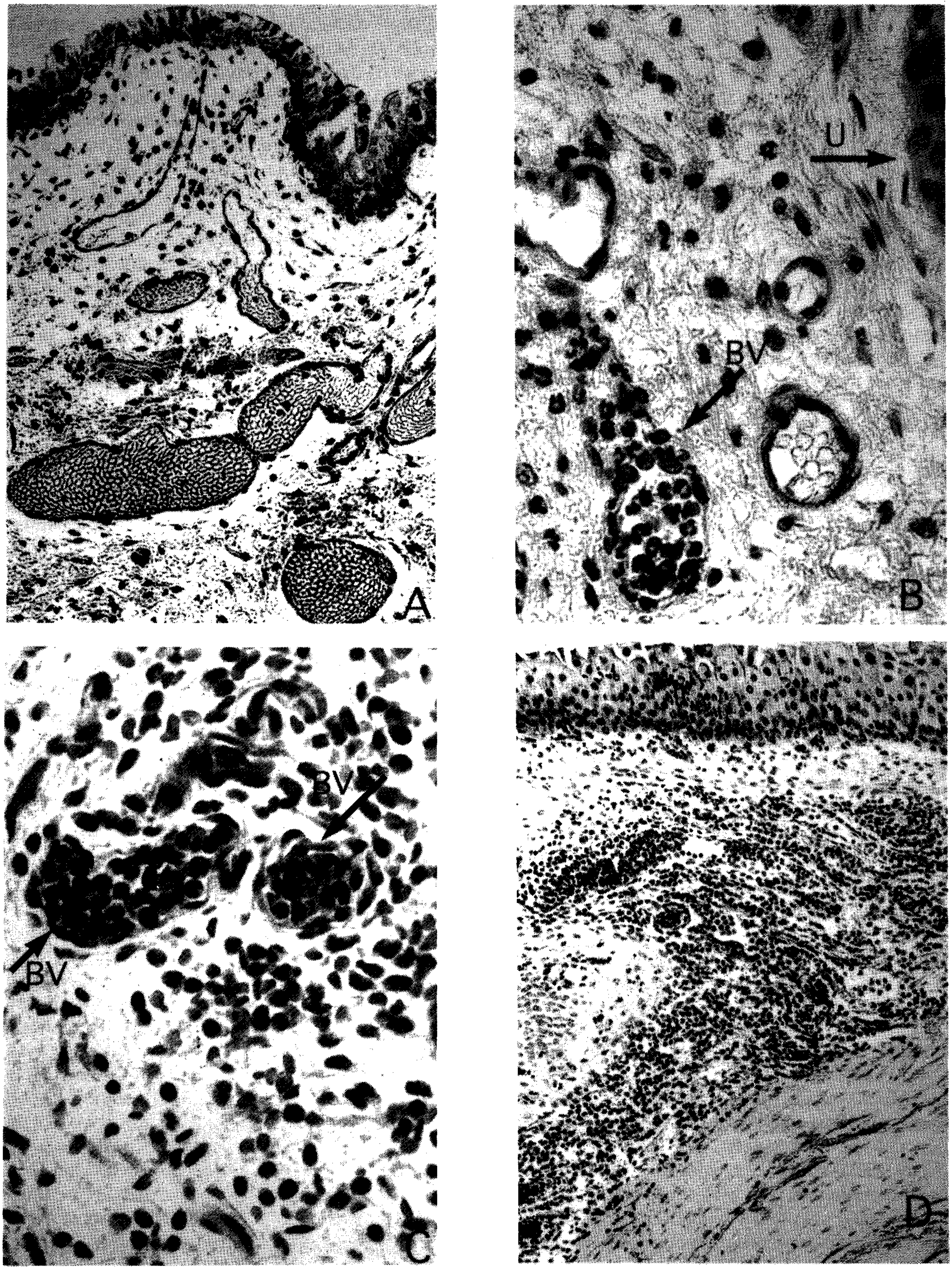

FIG. 2. These photomicrographs illustrate the progression of histological changes in the bladder wall after intravesical instillation of capsaicin 3 μM, SP 100 μM or ovalbumin 1 μg/ml (sensitized animals only). The progression of increasing severity of cellular infiltration was similar for these three groups, but capsaicin-treated bladders exhibited significantly more haemorrhage. Examples of OVA-treated bladders from sensitized animals are shown, because the changes are less obscured by haemorrhage. **A**, 30 min after challenge (approximately 200 ×); **B**, 8 h (approximately 400 ×); **C**, 12 h (approximately 600 ×); and **D**, 20 h after challenge (approximately 100 ×). Arrows indicate urothelium (U) or blood vessels (BV).

stimulating Evans blue extravasation in amounts most closely approximating that in response to 1 μg/ml OVA were chosen for study to allow comparison of the effects of these substances. Therefore, although extravasation of Evans blue dye induced by 3 μM capsaicin was significantly higher than that induced by 100 μM SP or 1 μg/ml OVA, this observation is a function of the relative concentrations of capsaicin, SP and OVA chosen to be studied.

*Leukocyte migration:* Histological changes in the bladder tissue after capsaicin or SP exposure (or intravesical instillation of OVA in sensitized guinea-pigs) appeared to follow a time course of increasing severity between 30 min and 20 h post-exposure, progressing from pronounced vasodilatation to marginalization of granulocytes, followed by diapdesis of leukocytes, initially restricted to the perivascular area and subsequently spreading through the submucosa and into the detrusor (Fig. 2). Eight hours after SP exposure, a profuse cellular migration within the submucosa near the urothelium was observed, and leukocytes were also present between muscle bundles of the detrusor. Leukocytes continued to migrate into these areas for the next 12 h, at which time the histological appearance of the tissues stabilized.

Intravesical saline caused minimal histological changes in the bladder wall (Fig. 3). However, sensory nerve stimulation by intravesical instillation of capsaicin induced intense vasodilatation, haemorrhage and leukocyte infiltration of the submucosa and detrusor muscle. Granulocytes, most of which were releasing their granules, were the predominant leukocyte observed in the wall of the guinea-pig bladder after capsaicin exposure, but lymphocytes were also present. Similar effects were observed after intravesical administration of substance P. Intravesical instillation of OVA had no effect on the histological appearance of the bladders of non-sensitized animals and caused less haemorrhage than SP or capsaicin exposure but a similar progression of other histological changes when instilled into the bladders of sensitized guinea-pigs.

*Inflammatory mediator release:* Substance P induced $PGD_2$ and LLI release from isolated sensitized and control bladder tissue; OVA had no effect on unsen sitized bladder tissue but stimulated $PGD_2$ and LLI release from sensitized bladder tissue (Fig. 4). Substance P stimulated release of relatively more prostaglandins and less leukotrienes than OVA exposure of sensitized bladder tissue. Increasing the concentration of either SP (to 1 000 μM) or OVA (to 100 μg/ml), or increasing incubation time up to 90 min had no effect on $PGD_2$ or LLI release (data not shown).

*Activation of sensory neurones by inflammatory mediators: In vitro* exposure of guinea-pig bladder tissue to various mediators of inflammation, including histamine, $LTC_4$ and $PGD_2$, stimulated SP release (Fig. 5). *In vitro* exposure of bladder tissue from sensitized guinea-pigs (but not from control animals) to OVA also stimulated SP release. SP release in response to these substances was compared with SP release in response to activation of sensory nerves with capsaicin and exposure of bladder tissue to a non-physiological, non-immunological stimulus (calcium ionophore). Calcium ionophore stimulated a comparatively low amount of SP release at this concentration, while capsaicin stimulated significant SP release, as expected.

### Discussion

The results of these experiments clearly demonstrate the effects of stimulation of sensory nerves on inflammation of the guinea-pig urinary bladder. Although the concentrations of SP evaluated in these experiments exceed expected tissue concentrations, the capacity of endogenous SP to produce similar results is confirmed by the response of the bladder to treatment with capsaicin. The concentrations of capsaicin, SP and OVA used in these experiments were selected to produce a similar degree of plasma extravasation as measured by tissue concentrations of Evans blue dye to allow direct comparison between the effects of these substances. The effects of SP and capsaicin appear to be concentration dependent, and lower concentrations used in preliminary studies caused primarily mild interstitial oedema and marginalization of leukocytes within the vasculature despite marked vasodilatation.

Using this guinea-pig model of antigen induced cystitis, the authors have previously described release of inflammatory mediators, including histamine, $PGD_2$ and leukotrienes, from the bladder subsequent to antigen sensitization and challenge.[21,22] Other investigators have reported that antigen sensitization results in alterations of micturition frequency, voiding volume and cystometrographic data in guinea-pigs, as well as increased uptake of intravesical $^{14}$C-urea.[26,27] The present experiments demonstrate that OVA sensitization and subsequent challenge of guinea-pig bladder causes release of SP, and that SP is released from naive and sensitized bladder tissue in response to histamine, $PGD_2$, $LTC_4$ and A-23587. These results indicate that SP is released in response to inflammatory mediators, and it cannot be determined from these experiments whether or not antigen sensitization and exposure directly stimulates SP release.

In a cyclophosophamide induced cystitis model in rats, Maggi *et al.* demonstrated that bladder hyperreflexia was mediated through stimulation of

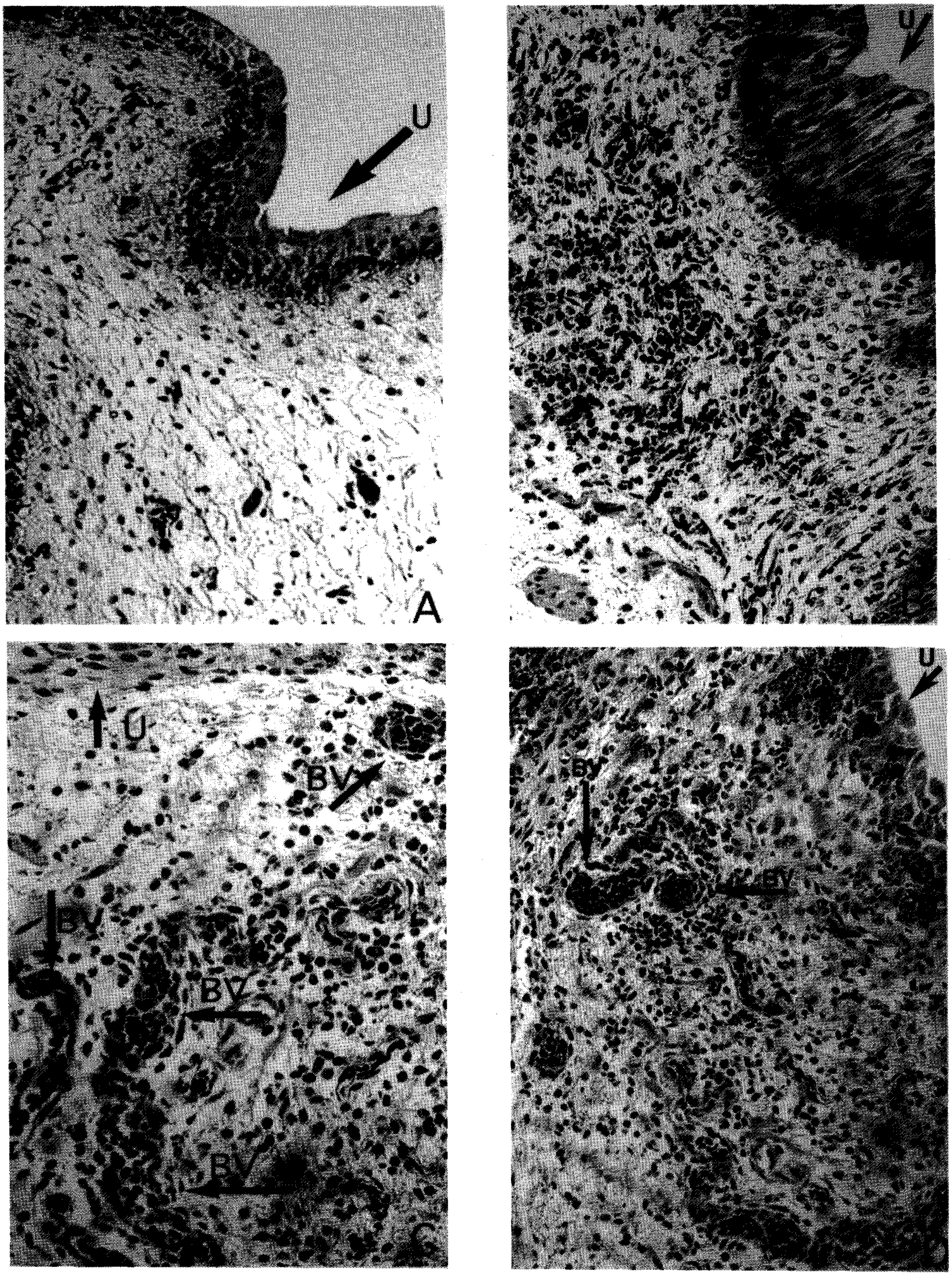

FIG. 3. Representative photomicrographs of guinea-pig bladders 20 h after *in vivo* exposure to saline (**A**, approximately 200 x); OVA (**B**, sensitized animals only, 1 μg/ml, approximately 200 x); substance P (**C**, 100 μM, approximately 400 x); or capsaicin (**D**, 3 μM, approximately 200 x). Arrows indicate the urothelium (U) or blood vessels (BV). Note marked vasodilatation induced by SP (**C**) and intense haemorrhage induced by capsaicin (**D**).

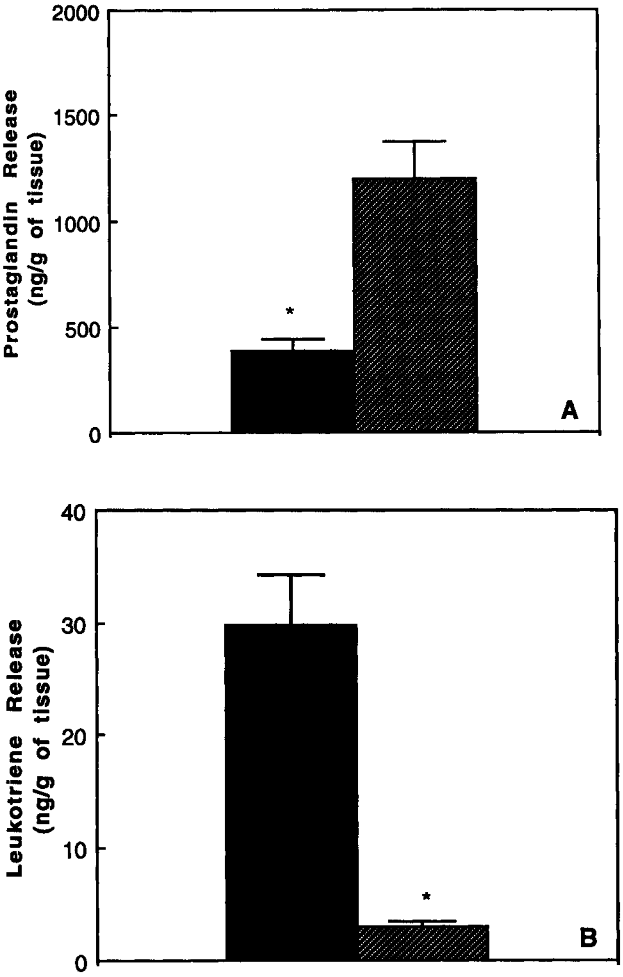

FIG. 4. *In vitro* release of prostaglandin $D_2$ (A) and leukotriene-like immunoreactivity (B) by guinea-pig bladders incubated with substance P (naive and sensitized animals) and ovalbumin (sensitized animals only). Substance P stimulated release of significantly more $PGD_2$, while ovalbumin stimulated release of significantly more leukotriene. ■, ovalbumin, 1 μg/ml; ▨, substance P, 100 μM.

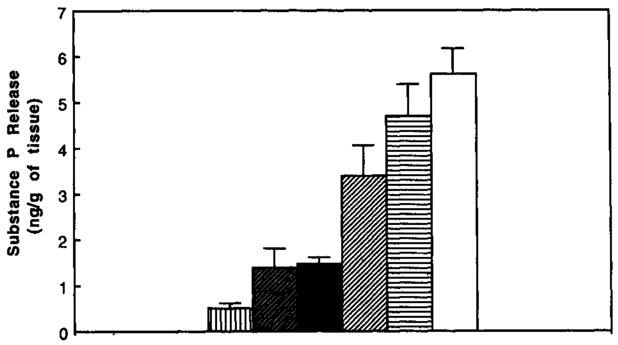

FIG. 5. *In vitro* release of substance P in response to various stimuli. At the concentrations of agonists used, capsaicin, histamine and $PGD_2$ stimulated release of comparable quantities of SP. Leukotriene $C_4$, ovalbumin (sensitized bladders only) and calcium ionophore A-23587 at the concentrations used stimulated release of smaller, but detectable, quantities of SP. ▥, A-23587, 100 μM; ▩, $LTC_4$, 100 μM; ■, ovalbumin, 1 μg/ml; ▨, $PGD_2$, 100 μM; ⊟, histamine, 100 μM; □, capsaicin, 3 μM.

unmyelinated afferent nerve fibres but that capsaicin pretreatment actually increased plasma extravasation as measured by Evans blue.[28] Bilateral removal of the pelvic ganglia almost eliminated plasma extravasation within the bladder wall in response to cyclophosphamide and suppressed enhancement of plasma extravasation by capsaicin pretreatment. The differences between this model and the present results may be explained in part by the authors' observation that cyclophosphamide-induced cystitis is not accompanied by a toxic effect on the afferent nerves of the bladder but may be accompanied by stimulation of the ganglia.

The calcium ionophore A-23587 is a non-immunologic secretagogue for mast cell products.[29] Although SP release in response to this compound was relatively small at the concentration used, this finding supports the capacity of mast cell degranulation to stimulate SP release. Degranulating mast cells release histamine, prostaglandins and leukotrienes, among other inflammatory mediators. It is apparent that, at least in the guinea-pig bladder, these compounds stimulate release of SP, most likely from unmyelinated afferent nerve fibres, which can then promote and amplify inflammation. These effects may be concentration dependent, and it is probable that SP release could be altered by varying the concentration of agonists used.

The capacity of SP to stimulate histamine release from mast cells has been demonstrated repeatedly, but sensitivity of mast cells to the effects of SP seems to be specific to the species and tissue of origin of the mast cells.[20,23,30] In separate studies, we have demonstrated SP-induced histamine release from guinea-pig bladder, and confirmed release of $PGD_2$ and leukotrienes from guinea-pig bladder in response to SP (unpublished data). SP and SP analogues have also been shown to stimulate release of leukotrienes and thromboxane $B_2$ from isolated, perfused rat heart.[30] Pretreatment of experimental animals with capsaicin or antagonists for the primary SP receptor (NK-1) prevents, or greatly reduces the severity of, neurogenic inflammation, while inhibition of neutral endopeptidases which degrade neuropeptides amplifies the effects of neurogenic inflammation.[10,31] Abelli *et al.*[18] demonstrated that SP administered intravenously to rats caused increased post-capillary venular permeability within the urinary bladder wall that was not exclusively $NK_1$-receptor-mediated. Yano *et al.* have suggested that SP-induced increased vascular permeability and inflammatory cell migration are mast cell-dependent phenomena.[32] While leukotrienes and other metabolites of arachidonic acid released in response to SP may come from a variety of sources, including mast cells, vascular endothelium and smooth muscle, it appears that mast cells are the sole source of histamine.[30,32] Neuropeptide-induced increased vascular permeability clearly appears to be in part due to interaction of SP with mast cells.

The role of mast cells in the pathogenesis of IC has been the subject of considerable debate. IC is characterized by chronic inflammation, and several reports suggest a role for increased mast cell numbers (and possibly activity) in the initiation and propagation of IC,[33,34] although others question whether or not mast cells are involved.[35] Participation of neuropeptides in the pathogenesis of IC may not rely solely on the presence and degranulation of mast cells. SP and CGRP also have the capacity to modify the function of other cells of the immune system.[9] SP acts as a chemoattractant and migratory stimulant for monocytes and neutrophils, and SP receptors have been identified on the surface of these cells.[36,37] SP receptors have also been identified on the surface of lymphocytes,[38] and functional receptors for CGRP have been characterized on lymphocytes and macrophages in mice and rats.[37,39,40] SP stimulates lymphocyte proliferation, migration and immunoglobulin production.[37,38] CGRP stimulates lymphocyte migration but may inhibit lymphocyte proliferation.[37,39] SP stimulates neutrophil antibody dependent, cell mediated cytotoxicity and may facilitate neutrophil production of toxic oxygen species.[41]

Several reports have described increased nerve density in the bladder wall in the presence of IC, including Hand in 1949[5] and more recent investigators.[6,7] Lasanen *et al.*[12] described a marked, extensive depletion of VIP, neuropeptide Y, and SP-immunoreactive nerve fibres after distension of the rat bladder wall. This depletion of SP-containing sensory nerves was reversible as early as 21 days later. A decrease in active sensory nerves or decreased concentrations of neurotransmitters may contribute to transient relief of pain provided IC patients by bladder distension. Increased SP activity was observed during the recovery phase in previously distended rat bladders; this may be related to neurogenic inflammation and resumption of clinical symptoms. Intravesical instillation of capsaicin in five patients with hypersensitivity disorders of the lower urinary tract provided temporary relief of symptoms in all patients, strongly suggesting participation of unmyelinated afferent nerve fibres in sensation of pain in the lower urinary tract.[42] Systemic administration of capsaicin to newborn rats decreased detrusor hyperreflexia and vascular permeability associated with xylene-induced cystitis.[19] In humans, local application of capsaicin has been shown to block the axon-flare response and the flare response to intradermal allergen and vasosactive inflammatory mediators.[43]

The results of the experiments described suggest that bladder inflammation in the absence of infection may at least in part be due to initiation of a cyclic sequence of events which entails release of inflammatory mediators by a variety of stimuli which subsequently themselves stimulate, directly or indirectly, release of neuropeptides. Neuropeptides, particularly SP, contribute to continued release of inflammatory mediators, perpetuating and amplifying inflammation. Chronic bladder inflammation may thus be the result of a variety of initiating factors. This also suggests that, to be effective, therapeutic interventions should be directed at more than one phase of this cyclic process.

### References

1. Hanno PM, Levin RM, Monson FC, *et al.* Diagnosis of interstitial cystitis. *J Urol* 1990; **143**: 278–281.
2. Kruger JM, Osborne CA, Goyal SM, *et al.* Clinical evaluation of cats with lower urinary tract disease. *J Am Vet Med Assoc* 1991; **199**: 211–216.
3. Holm-Bentzen M, Jacobsen F, Nerstrom B, *et al.* Painful bladder disease: clinical and pathoanatomical differences in 115 patients. *J Urol* 1987; **138**: 500–502.
4. Sant GR. Interstitial cystitis: pathophysiology, clinical evaluation, and treatment. In Rous S, ed. *Urology Annual*, Norwalk, CT; Appleton & Lange, 1989; 171–196.
5. Hand JR. Interstitial cystitis: report of 223 cases (204 women and 19 men). *J Urol* 1949; **61**: 291–310.
6. Christmas TJ, Rode J, Chapple CR, Milroy EF, Turner-Warwick RT. Nerve fibre proliferation in interstitial cystitis. *Virchows Arch A Pathol Anat Histopathol* 1990; **416**: 447–451.
7. Lundeberg T, Liedberg H, Nordling L, Theodorsson E, Owzarski A, Ekman P. Interstitial cystitis: correlation with nerve fibres, mast cells and histamine. *Br J Urol* 1993; **71**: 427–429.
8. Kowalski ML, Sliwinska-Kowalska M, Kaliner MA. Neurogenic inflammation, vascular permeability, and mast cells. II. Additional evidence indicating that mast cells are not involved in neurogenic inflammation. *J Immunol* 1990; **145**: 1214–1221.
9. Sharkey KA. Substance P and calcitonin gene-related peptide (CGRP) in gastrointestinal inflammation. *Ann NY Acad Sci* 1992; **664**: 425–442.
10. Yonehara N, Imai Y, Shibutani T, Inoki R. Participation of substance P in inflammatory responses. *Adv Exp Med Biol* 1989; **247B**: 529–534.
11. Gu J, Blank MA, Huang WM, *et al.* Peptide-containing nerves in human urinary bladder. *Urology* 1984; **24**: 353–357.
12. Lasanen LT, Tammela TL, Liesi P, Waris T, Polak JM. The effect of acute distension on vasoactive intestinal peptide (VIP), neuropeptide (NPY), and substance P (SP) immunoreactive nerves in the female rat urinary bladder. *Urol Res* 1992; **20**: 259–263.
13. Maggi CA, Patacchini R, Sanicioli P, Giuliani S. Tachykinin antagonists and capsaicin-induced contractions of the rat isolated urinary bladder: evidence for tachykinin-mediated cotransmission. *Br J Pharmacol* 1991; **103**: 1535–1541.
14. Maggi CA. The dual function of capsaicin-sensitive sensory nerves in the bladder and urethra. *Ciba Found Symp* 1990; **151**: 77–90.
15. Lundberg JM, Brodin E, Hua X, Saria A. Vascular permeability changes and smooth muscle contraction in relation to capsaicin-sensitive substance P afferents in the guinea-pig. *Acta Physiol Scand* 1984; **120**: 217–227.
16. Brokaw JJ, White GW. Calcitonin gene-related peptide potentiates substance P-induced plasma extravasation in the rat trachea. *Lung* 1992; **170**: 85–93.
17. Abelli L, Nappi F, Perretti F, Maggi CA, Manzini S, Giachetti A. Microvascular leakage induced by substance P in the rat urinary bladder: involvement of cyclo-oxygenase metabolites of arachidonic acid. *J Auton Pharmacol* 1992; **12**: 269–276.
18. Abelli L, Somma V, Maggi CA, *et al.* Effects of tachykinins and selective tachykinin receptor antagonists on vascular permeability in the rat lower urinary tract: evidence for the involvement of NK-1 receptors. *J Auton Pharmacol* 1989; **9**: 253–263.
19. Maggi CA, Abelli L, Giuliani S, *et al.* The contribution of sensory nerves to xylene-induced cystitis in rats. *Neuroscience* 1988; **26**: 709–723.
20. Church MK, Lowman MA, Robinson C, Holgate ST, Benyon RC. Interaction of neuropeptides with human mast cells. *Int Arch Allergy Appl Immunol* 1989; **88**: 70–78.
21. Christensen MM, Keith I, Rhodes PR, *et al.* A guinea pig model for study of bladder contraction. *J Urol* 1990; **144**: 1293–1300.
22. Saban R, Christensen M, Keith I, *et al.* Experimental model for the study of bladder mast cell degranulation and smooth muscle contraction. *Sem Urol* 1991; **9**: 88–101.
23. Undem BJ, Pickett WC, Lichtenstein LM, Adams GK III. The effect of indomethacin on immunologic release of histamine and sulfidopeptide leukotrienes from human bronchus and lung parenchyma. *Am Rev Respir Dis* 1987; **136**: 1183–1187.
24. Aharony D, Dobson P, Berstein PR, Kusner EJ, Krell RD, Smith JB. Determination of SRS-A release from guinea pig lungs by a radioimmunoassay. *Biochem Biophys Res Comm* 1983; **117**: 574–579.
25. Bernard R, ed. *Fundamentals of Biostatistics*. Boston, MA: PWS Publishers, 1990; 442–450.
26. Kim YS, Longhurst PA, Wein AJ, Levin RM. Effects of sensitization on female guinea pig urinary bladder function: *in vivo* and *in vitro* studies. *J Urol* 1991; **146**: 454–457.
27. Kim YS, Levin RM, Wein AJ, Longhurst PA. Effects of sensitization on the permeability of the urothelium in the guinea pig urinary bladder. *J Urol* 1992; **147**: 270–273.
28. Maggi CA, Lecci A, Santicioli P, Del Bianco E, Giuliani S. Cyclophosphamide cystitis in rats: involvement of capsaicin-sensitive primary afferents. *J Auton Nerv Syst* 1992; **38**: 201–208.

29. Vliagoftis H, Dimitriadou V, Boucher W, *et al.* Estradiol augments while tamoxifen inhibits rat mast cell secretion. *Int Arch Allergy Immunol* 1992; **98**: 398–409.
30. Assem ESK, Ghanem NS, Abdullah NA, Repke H, Foreman JC, Hayes NA. Substance P and arg-pro-lys-pro-NH-$C_{12}$-$H_{25}$-induced mediator release from different mast cell subtypes of rat and guinea-pig. *Immunopharmacology* 1989; **17**: 119–128.
31. Nadel JA. Neurogenic inflammation in airways and its modulation by peptidases. *Ann NY Acad Sci* 1992; **664**: 408–414.
32. Yano H, Wershil BK, Arizono N, Galli SJ. Substance P-induced augmentation of cutaneous vascular permeability and granulocyte infiltration in mice is mast cell dependent. *J Clin Invest* 1989; **84**: 1276–1286.
33. Nielsen KK, Kroman-Andersen B, Steven K, Hald T. Failure of combined supratrigonal cystectomy and Mainz ileocecocystoplasty in intractible interstitial cystitis: is histology and mast cell count a reliable predictor for the outcome of surgery? *J Urol* 1990; **144**: 255–258.
34. Theoharides TC, Flarris N, Cronin CT, Ucci A, Meares E. Mast cell activation in sterile bladder and prostate inflammation. *Int Arch Allergy Appl Immunol* 1990; **92**: 281–286.
35. MacDermott JP, Charpied GL, Tesluk H, Stone AR. Can histological assessment predict the outcome in interstitial cystitis? *Br J Urol* 1991; **67**: 44–47.
36. Haines KA, Kolasinski SL, Cronstein BN, Reibman J, Gold LI, Weissmann G. Chemoattraction of neutrophils by substance P and transforming growth factor-beta 1 is inadequately explained by current models of lipid remodeling. *J Immunol* 1993; **151**: 1491–1499.
37. McGillis JP, Mitsuhashi M, Payan DG. Immunomodulation by tachykinin neuropeptides. *Ann NY Acad Sci* 1990; **594**: 85–94.
38. Pascual DW, Bost KL, Xu-Amano J, Kiyono H, McGhee JR. The cytokine-like action of substance P upon B cell differentiation. *Reg Immunol* 1992; **4**: 100–104.
39. Umeda Y, Arisawa M. Characterization of the calcitonin gene-related peptide receptor in mouse T lymphocytes. *Neuropeptides* 1989; **14**: 237–242.
40. Abello J, Kaiserlian D, Cuber JC, Revillard JP, Chayvialle JA. Characterization of calcitonin gene-related peptide receptors and adenylate cyclase response in the murine macrophage cell line P388 D1. *Neuropeptides* 1991; **19**: 43–49.
41. Wozniak A, McLennan G, Betts WH, Murphy GA, Scicchitano R. Activation of human neutrophils by substance P: effect on FMLP-stimulated oxidative and arachidonic acid metabolism and on antibody-dependent cell-mediated cytotoxicity. *Immunology* 1989; **68**: 359–364.
42. Maggi CA, Barbanti G, Santicioli P, *et al.* Cystometric evidence that capsaicin-sensitive nerves modulate the afferent branch of micturition reflex in humans. *J Urol* 1989; **142**: 150–154.
43. Lundblad L, Lundberg L, Anggard A, Zetterstrom O. Capsaicin pretreatment inhibits the flare component of the cutaneous allergic reaction in man. *Eur J Pharmacol* 1985; **113**: 461–462.

ACKNOWLEDGEMENTS. This work was supported by grant #1R01DK42897-C1A2 from National Institutes of Health.

The authors acknowledge the contribution of Joel Armstrong from The Radionuclide Laboratory, School of Veterinary Medicine, University of Wisconsin–Madison for assistance in the substance P radioimmunoassays and Kermit Groothuis for assistance with tissues processing for histology.

**Received 17 January 1994; accepted in revised form 15 February 1994**
